# Promising Antileishmanial Activity of *Micromeria nervosa* Essential Oil: In Vitro and In Silico Studies

**DOI:** 10.3390/molecules29081876

**Published:** 2024-04-19

**Authors:** Rym Essid, Sarra Kefi, Bilel Damergi, Ghassen Abid, Nadia Fares, Selim Jallouli, Islem Abid, Dina Hussein, Olfa Tabbene, Ferid Limam

**Affiliations:** 1 Laboratory of Bioactive Substances, Biotechnology Center of Borj Cedria, BP 901, Hammam-Lif 2050, Tunisia; kefi.sarra@gmail.com (S.K.); nadia.fares@gmail.com (N.F.);; 2 University of Tunis-El Manar, Campus Universitaire Farhat Hached, BP-94 Rommana, Tunis 1068, Tunisia; 3 Laboratory of Legumes and Sustainable Agro-Systems, Centre of Biotechnology of Borj Cedria, Hammam-Lif 2050, Tunisia; 4 Center of Excellence in Biotechnology Research, College of Applied Medical Sciences, King Saud University, Riyadh 11495, Saudi Arabia; 5 Department of Chemistry, College of Sciences and Health, Cleveland State University, Cleveland, OH 44115, USA; dinahussein12@gmail.com

**Keywords:** leishmanicidal activity, cytotoxicity, *Micromeria nervosa* EO, sterol and thiol pathways, molecular docking

## Abstract

The present study aimed to evaluate the leishmanicidal potential of the essential oil (EO) of *Micromeria* (*M.*) *nervosa* and to investigate its molecular mechanism of action by qPCR. Furthermore, in silicointeraction study of the major *M. nervosa* EO compounds with the enzyme cytochrome P450 sterol 14α-demethylase (CYP51) was also performed. *M. nervosa* EO was analyzed by gas chromatography-mass spectrometry (GC-MS). Results showed that α-pinene (26.44%), *t*-cadinol (26.27%), caryophyllene Oxide (7.73 ± 1.04%), and α-Cadinene (3.79 ± 0.12%) are the major compounds of *M. nervosa* EO. However, limited antioxidant activity was observed, as this EO was ineffective in neutralizing DPPH free radicals and in inhibiting β-carotene bleaching. Interestingly, it displayed effective leishmanicidal potential against promastigote (IC_50_ of 6.79 and 5.25 μg/mL) and amastigote (IC_50_ of 8.04 and 7.32 μg/mL) forms of *leishmania (L.) infantum* and *L. major*, respectively. Molecular mechanism investigation showed that *M. nervosa* EO displayed potent inhibition on the thiol regulatory pathway. Furthermore, a docking study of the main components of the EO with cytochrome P450 sterol 14α-demethylase (CYP51) enzyme revealed that *t*-cadinol exhibited the best binding energy values (−7.5 kcal/mol), followed by α-cadinene (−7.3 kcal/mol) and caryophyllene oxide (−7 kcal/mol). These values were notably higher than that of the conventional drug fluconazole showing weaker binding energy (−6.9 kcal/mol). These results suggest that *M. nervosa* EO could serve as a potent and promising candidate for the development of alternative antileishmanial agent in the treatment of leishmaniasis.

## 1. Introduction

Leishmaniasis is a complex parasitic infection caused by the protozoan *Leishmania* parasite. It is recognized as a significant neglected tropical disease. Globally, more than 350 million individuals are affected worldwide, with approximately 2 million new cases reported annually in developing countries. The increasing number of infection cases is related to the limited effectiveness of conventional treatments, such as meglumine antimoniate and pentamidine isethionate, and the absence of a vaccine [1]. Although alternative therapies such as miltefosine and paromomycin, as well as new formulations of older drugs such as amphotericin B (either in lipid solution or by lipid pulse technique), none of them provide adequate treatment for the disease. Furthermore, these treatments are associated with notable side effects, high costs, and most importantly, the potential to develop drug resistance [2]. This serves as an escape mechanism for the parasite and enhances its chances of survival in the parasitophorous vacuole. 

The challenges in effectively managing leishmaniasis highlight the urgent need for improved treatment options that are more effective, affordable, and have fewer side effects. Continuous research and development efforts are critical to address these issues and provide better solutions for the prevention and treatment of this infection. 

In recent years, plant extracts have received much attention as potential sources of drugs to treat leishmaniasis [3]. Under such circumstances, medicinal and aromatic plants have recently emerged as an interesting therapeutic alternative for healthcare and wellness [4]. Bioactive compounds are naturally produced by these plants and serve as a defense mechanism against environmental threats. Particular emphasis is placed on essential oils. These compounds have gradually attracted attention in a number of fields including cosmetics and therapeutic applications due to their medical properties [5]. 

The Lamiaceae family, also known as the mint family, is a widespread group of flowering plants. It includes a variety of plant species such as herbs such as mint, sage, rosemary, thyme oregano, and lavender. This family is quite extensive; it comprises around 7000 species found in parts of the world. Additionally, it is characterized by square stems, opposite leaves, and a distinctive aromatic scent derived from the presence of EO found in the leaves and flowers. These EOs contribute not only to the pleasant fragrance of Lamiaceae plants but also to their therapeutic properties [6]. Among this family, *Micromeria nervosa*, a highly valued medicinal and aromatic herb is known for its various therapeutic properties in traditional medicine. This species has been widely used for its fragrant nature. The leaves of *M. nervosa* are commonly used topically to treat skin infections, while a decoction made from the leaves can be used to treat respiratory, stomach, and intestinal problems. Additionally, the plant exhibits antiseptic, tonic, cardiotonic, and hypotensive properties [7]. For thousands of years, people in Mediterranean regions have relied on this plant as a source of medicine [8,9]. Previous research conducted on *M. nervosa* has identified several molecules with diverse biological activities such as Furan-sesquiterpene alcohol (Micromeriol), 5-β-cholestane (Nervosane), and Triterpenioc acids (Oleanolic acid and Ursolic acid) through phytochemical studies [10]. These findings enhance the understanding of the plant’s therapeutic potential and highlight its potential in traditional and alternative medicine. 

Recently, the *Leishmania* sterol biosynthesis pathway has been regarded as an attractive target for the development of selective drugs. A crucial enzyme in this pathway is the sterol 14α-demethylase, known as CYP51, which plays a key role in the conversion of lanosterol to 4,4-dimethylcholesta-8(14),24-dien-3β-ol in the *Leishmania* parasite [11]. Several sterol inhibitors, including azoles, allylamines, and statins, have been shown to effectively inhibit CYP51 activity, leading to sterol depletion in *Leishmania* parasites and consequently affecting the fluidity and integrity of the fungal membrane. Therefore, the development of new drugs specifically targeting CYP51-mediated sterol biosynthesis in *Leishmania* parasites has promising potential as an effective medication strategy for leishmaniasis [12]. 

Additionally, trypanothione reductase (TR) is widely recognized as a highly promising target for novel drug discovery against leishmaniasis. This enzyme plays a crucial role in the survival of the parasite in the human host by facilitating the reduction of trypanothione. Trypanothione is an important molecule used by the tryparedoxin/tryparedoxin peroxidase system to counteract the detrimental effects of hydrogen peroxide generated by host macrophages during infection. Consequently, inhibition of TR could disrupt this essential defence mechanism employed by the parasite and potentially lead to the development of effective treatments for leishmaniasis. This serves as an escape mechanism for the parasite and enhances its chances of survival in the parasitophorous vacuole. 

The objective of the present study is to evaluate the antileishmanial activity of *M. nervosa* (Desf.) Benth. EO against the amastigote and promastigote forms of *L. major* and *L. infantum* and to study its molecular mechanism of action. 

## 2. Results

### 2.1. Extraction Yield and GC-MS Analysis of M. nervosa EO

The extraction of EO from the leaves of *M. nervosa* plants gives a yield of 0.28%. GC-MS analysis of *M. nervosa* EO shows the identification of 29 compounds (Table 1). Alpha-pinene (26.44%) and *t*-cadinol (26.27%) are the main constituents, followed by caryophyllene oxide (7.73%). Other compounds have also been identified such as alfa cadinene (3.79%), alfa bisabolol (3.77%), *d*-l-limonene (3.25%), and l-linalool (2.08%) as well as minor constituents, ranging from 1% to 0.1%. These results showed that *M. nervosa* EO contains many classes of terpene compounds, with monoterpenes (41.26%) and oxygenated sesquiterpenes (38.75%) being the most prevalent. 

### 2.2. Antioxidant Activity of M. nervosa EO

The assessment of the antioxidant activity of *M. nervosa* EO involved DPPH radical scavenging and β-carotene bleaching assays (Table 2). The antioxidant potential of *M. nervosa* EO is relatively low compared to the synthetic antioxidant BHT. Particularly, *M. nervosa* EO displayed noteworthy DPPH radical scavenging activity, showing an IC_50_ value of 933.12 μg/mL. Furthermore, its efficacy in beta-carotene bleaching was limited (IC_50_ = 489.45 μg/mL). 

### 2.3. Antileishmanial Activity and Cytotoxicity of M. nervosa EO

#### 2.3.1. Antipromastigote Activity

*M. nervosa* EO demonstrated notable inhibitory activity displaying high selectivity toward both promastigote and amastigote forms of leishmania. The detailed results shown in Table 3, demonstrate that *M. nervosa* EO exhibited a reduction in parasite growth for the species tested with IC_50_ values of 6.79 μg/mL and 5.24 μg/mL against *L. major* and *L. infantum*, respectively. Importantly, *M. nervosa* EO did not show toxic effects against Raw 264.7 macrophages, as indicated by the respective selectivity index (SI) values of 11.82 and 15.31 on *L. major* and *L. infantum*. These results suggest that *M. nervosa* EO has a higher leishmanicidal potential against *L. major* and *L. infantum* species, and low cytotoxicity toward Raw 264.7 macrophage cells. 

#### 2.3.2. Antiamastigote Activity

The antiamastigote assay showed that *M. nervosa* EO exhibited high activity against both *L. major* and *L. infantum*, with IC_50_ values of 8.04 μg/mL and 7.32 μg/mL, respectively. As shown in Table 4, the specificity index obtained (situated between 0.4 and 2) shows that *M. nervosa* EO is active against both promastigote and amastigote forms. 

### 2.4. Molecular Mechanism of Action of M. nervosa EO

**Figure 1:**
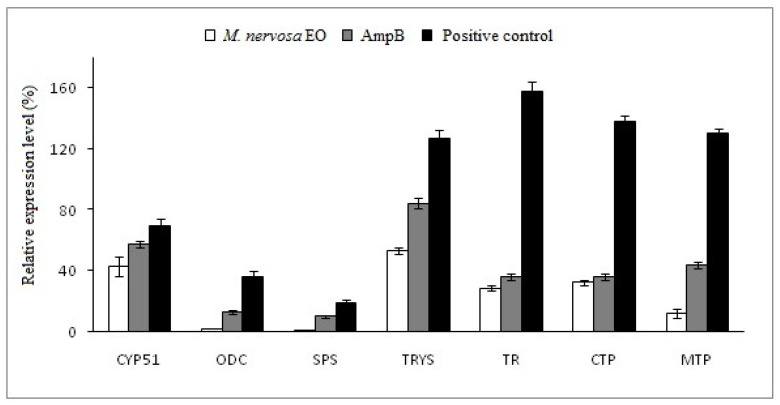
Relative expression level of genes involved in sterol biosynthesis and thiol metabolism treated with *M. nervosa* EO on *L. major* promastigotes. Negative control: untreated parasites, AmpB: amphotericin B. The relative expression of CYP51, ODC, SPS, TRYS, TR, CTP, and MTP genes was quantified by qPCR using 18SrRNA housekeeping genes as normalization control.

The underlying molecular mechanism associated with *M. nervosa* EO on *L. major* promastigotes was investigated, revealing its impact on two major regulatory pathways: sterol biosynthesis and thiol metabolism. As shown in Figure 1, the thiol pathway was significantly affected by *M. nervosa* EO treatment, leading to repression of gene expression at multiple levels. Notably, the ODC gene exhibited a 15.22-fold reduction, while SPS, TS, TR, CTP, and MTP genes displayed reductions of 16.97-fold, 2.37-fold, 5.52-fold, 4.26-fold, and 10.67-fold, respectively. Amphotericin B (AmpB) causes greater repression of the ODC, SPS, and MTP genes by 2.78-fold, 1.83-fold, and 2.96-fold, respectively, compared to negative control (untreated parasites). However, treatment by *M. nervosa* EO led to a lower reduction (1.6-fold) in CYP51 gene expression compared to untreated parasites. Interestingly, the expression level genes in the leishmanial strain after treatment with AmpB remained comparable to that in untreated leishmaniasis, suggesting resistance to this drug. 

These findings highlight the specific molecular effects of *M. nervosa* EO on *L. major* promastigotes, targeting different genetic pathways involved in sterol biosynthesis and thiol metabolism. 

### 2.5. Molecular Docking Analysis

**Figure 2:**
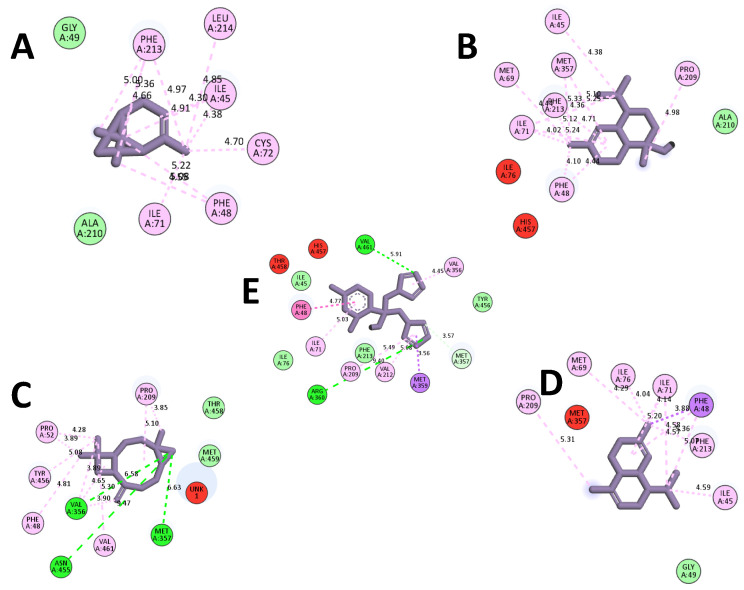
Docked poses of CYP51 with (**A**) α-Pinene, (**B**) *t*-Cadinol, (**C**) Caryophyllene Oxide, (**D**) α-Cadinene (**E**) Fluconazole. The structural graphics were generated using Discovery Studio 2021.

The molecular docking process facilitates the examination of interactions involving major EO compounds of *M. nervosa* including α-Pinene, *t*-Cadinol, α-Cadinene, and Caryophyllene oxide, with the active sites of the cytochrome P450 enzyme sterol 14α-demethylase (CYP51) from *Leishmania infantum* (Figure S1). The docking interactions of the target proteins with the ligands were presented in Figure 2, and the corresponding data were represented in terms of binding energy (∆G) in kcal mol^−1^ for all the studied molecules (Table 5). 

The predicted interaction of *t*-Cadinol was anchored in the active site of sterol 14-alpha demethylase (CYP51) and showed the best binding energy of −7.50 Kcal/mol involving eight alkyl interactions with ILE45, PHE48, MET69, ILE71, PRO209, PHE213, and MET357; four Pi-alkyl interactions with PHE48, ILE71, PHE213, and MET357; and one Van der Waals interaction with ALA. 210. After that, α-Cadinene occupies the active site of CYP51 with a low binding energy of −7.30 Kcal/mol involving eight alkyl interactions with ILE45, PHE48, MET69, ILE71, ILE76, PRO209, and PHE213; two Pi-alkyl interactions with PHE48 and ILE71; one Pi-Sigma interaction with PHE48; and one Van der Waals interaction with GLY49. 

**Figure 3:**
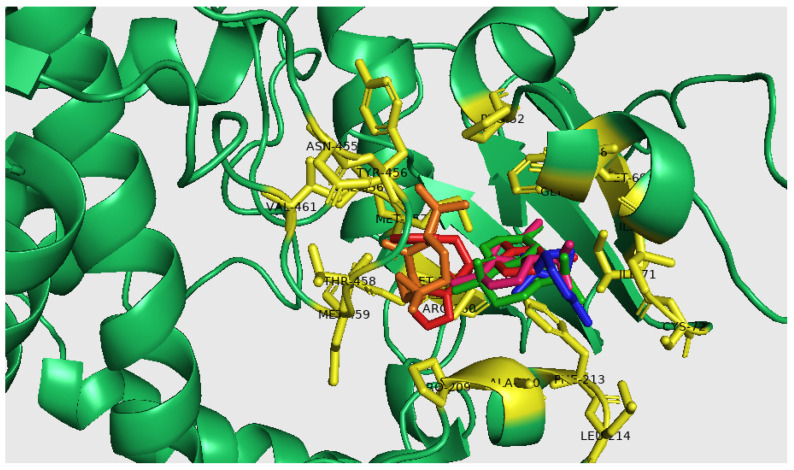
Docked poses of CYP51 with: Fluconazole (Red), α-Pinene (Blue), *t*-Cadinol (Green), caryophyllene Oxide (Orange), α-Cadinene (Pink). The structural graphics were generated using PyMOL(TM) 2.5. 4.

While caryophyllene oxide showed a good binding energy of −7.00 Kcal/mol involving three conventional hydrogen bonds with VAL356, MET357, and ASN455; seven alkyl bonds with PHE48, PRO52, PRO209, VAL356, TYR456, and VAL461; two with Pi-alkyl PRO209 and VAL356; and two Van der Waals bonds with THR458 and MET459. These interactions were greater than those obtained with the conventional CYP51 substrate “Fluconazole” showing a binding energy of −6.90 Kcal mol^−1^. The lowest binding energy was found with α-Pinene (−5.50 kcal mol^−1^) and involved nine Alkyl interactions with ILE45, PHE48, ILE71, CYS72, PHE213, and LEU214; three Pi-Alkyl bonds with ILE45, PHE48, and PHE213; and two Van der Waals interactions with GLY49 and ALA210. The docked poses of CYP51 with Fluconazole and major *M. nervosa* EO compounds were reported in Figure 3. The main interaction occurred through coordination with heme, accompanied by numerous hydrophobic interactions involving a variety of amino acid residues in diverse orientations. 

## 3. Discussion

In recent years, a significant global trend has emerged, with approximately 80% of people worldwide turning to herbal medicines and supplements as their primary healthcare solution [13]. The growing demand for herbal remedies is driving new research and advancements in drug development [14]. Notably, several active ingredients are actually derived from medicinal plants, which have proven crucial in drug discovery and development [15]. It is therefore essential to conduct ongoing research on plants to identify potential candidates that could serve as safer and more efficient agents in the future. 

*Micromeria nervosa* has shown promising therapeutic properties, and further exploitation of its essential oils could lead to further discoveries. According to our study, the essential oil yield of *M. nervosa* is relatively low (0.28%) compared to other species in the same genus, which typically exceed 0.5% due to their aromatic properties [16]. Other species, such as *M. dalmatica* (1.11%), *M. pulegium* (1.0%), *M. thymifolia* (0.99%), and *M. albanica* (0.88%), have been reported to have higher yields [17,18]. On the other hand, lower essential oil yields were observed in other species such as *M. debilis* (0.045%) and *M. inodora* (0.1%) [19,20,21]. It is important to acknowledge that the chemical composition and quantity of EO may vary depending on several factors such as extraction techniques, geographic location, and environmental conditions. 

In fact, the chemical composition of *M. nervosa* EO showed a similar distribution of compounds as other related species such as *M. cristata* (67%), *M. juliana* EOs (64.3%), *M. hortensis* (69.02%), *M. thymifolia* (70%), and *M. cilicica* (95%) [16,18,22,23,24]. 

However, different chemical compositions were observed as reported by [19], who identified 24 components including Elshatiza ketone, Cadalene, Oxo-Ylangene, Khusinol acetate, Borneol, Patchouli alcohol, and Carvenone. In addition, it was found that *M. nervosa* EO from Palestine is rich in carvacrol and thymol [7]. In Anatolia, *M. nervosa* EO exhibited richness in β-caryophyllene and caryophyllene oxide [25]. Additionally, the major components in Egyptian *M. nervosa* EO were cadalene (8.8%) and elsholtiza ketone (8.2%) [26]. 

Furthermore, the chemical composition of EOs can vary significantly among different *Micromeria* species. Pulegone is the major compound of *M. capitellata* EO (80%), *M. cilicica* EO (66.55%), *M. thymifolia* EO (32.81%), and *M. barbata* (20.19%) [18,27,28]. While piperitone oxide emerges as the major compound in *M. dalmatica* and *M. albanica* (41.77% and 38.73%, respectively) [17]. These variations in chemical composition are likely linked to the plant’s origin, which is influenced by the different bioclimatic conditions in which each species grows [29]. 

*M. nervosa* EO is characterized as a α-pinene and *t*-cadinol chemotype. α-pinene, in particular, is recognized for its antimicrobial activity [30], as well as its antioxidant and anticancer potential [31]. In contraste, *t*-cadinol has significant antifungal properties [32,33]. These chemical constituents contribute to the overall beneficial effects and potential therapeutic applications of *M. nervosa* EO. 

In the present study, *M. nervosa* EO showing moderate antioxidant activity may be attributed to the interaction between different constituents present in the EO [34]. Moreover, some organic compounds in the EO may show reduced solubility in the alcoholic solution used for the DPPH assay [35], which could further compromise its antioxidant capabilities. According to previous studies, EO from *M. nervosa* has the ability to reduce the stable free radical DPPH with an IC_50_ of 85 μg/mL due to its high content of oxygenated monoterpenes and oxygenated sesquiterpenes [19]. 

In addition, *M. nervosa* EO exhibited significant antileishmanial activity against promastigote and amastigote forms of *L. major* and *L. infantum* strains. The observed activity is likely attributable to major compounds, such as α-pinene, a monoterpene known for its potent leishmanicidal effects against promastigote forms of *L. major* and *L. infantum* with IC_50_ values of 17.6 and 19.8 μg/mL, respectively [36]. Likewise, α-pinene was reported to have an IC_50_ of 15.6 μg/mL against the amastigote form of *Leishmania amazonensis* [37]. Furthermore, *M. nervosa* EO showed no cytotoxic effects on Raw 264.7 macrophages. 

The second major compound, *t*-cadinol, has not yet been tested for leishmanicidal activity, although it has been identified as an important constituent of some EOs with anti-Leishmania potency [2]. The antiparasitic activity of this sesquiterpene alcohol has been demonstrated against *Trypanosoma* (T) *cruzi*, with IC_50_ values of 18 μM for trypomastigotes and 15 μM for amastigotes. Furthermore, *t*-cadinol showed no cytotoxicity against mammalian cells (SI>12) [33]. *t*-cadinol has been reported to induce mitochondrial deficiency, leading to hyperpolarization of membrane potential and decreased levels of reactive oxygen species [33]. A synergistic effect between α-pinene and *t*-cadinol could be suggested, even if they exhibit different mechanisms of action. 

In addition, minor compounds such as caryophyllene oxide and α-cadinene may contribute to significant leishmanicidal activity through synergistic effects. Therefore, the antileishmanial effect of *M. nervosa* EO appears to be greater than that of each individual compound. Caryophyllene oxide, a natural bicyclic sesquiterpene oxide found in various EOs, has demonstrated notable leishmanicidal activity against *L. amazonensis* promastigotes (IC_50_ = 4.90 μg/mL) and amastigotes (IC_50_ = 4.04 μg/mL) [38]. Furthermore, it exhibits significant in vitro antileishmanial activity against *L. infantum* amastigotes [2]. It acts within the mitochondria by partially inhibiting the electron transport chain in leishmania [39]. 

Moreover, it is important to note that the antileishmanial activity of α-cadinene lacks sufficient documentation. The precise mechanism of action remains incompletely understood, emphasizing the necessity for additional studies to elucidate the specific pathways involved. This highlights the complexity of EO composition and the importance of investigating how different compounds interact and contribute to their biological activities [40]. The synergy between compounds present in natural products can offer promising prospects for the development of effective and safe treatments against *Leishmania* infections [41].

Despite the limited understanding of their precise mechanism of action, some authors suggest that EOs with anti-leishmanial properties may offer an alternative therapeutic option as they exhibit non-toxic effects on host cells [37]. Additionally, other studies have demonstrated the efficacy of EOs against leishmaniasis and illustrated their non-toxic nature in animal models [42]. Furthermore, EOs exhibit strong anti-insecticide activities, which can help control sandfly bites and potentially limit the spread of the disease [14]. 

However, further research is essential to fully understand the mechanisms underlying the notable leishmanicidal activity of EOs and their potential as a natural remedy against *Leishmania* parasites. Other studies highlighted the action of *Chenopodium ambrosioides* EO at the mitochondrial level [39]. Plant EOs, as well as their major compounds such as ascaridole, carvacrol, and caryophyllene oxide, have demonstrated inhibitory effects on electron transport in the mitochondria of *L. tarentolae* promastigotes, leading to disruptions of mitochondrial functions [39]. More recently, enzymes associated with parasite metabolic pathways have emerged as promising targets for the development of novel antiparasitic compounds. This includes enzymes linked to sterol, trypanothione, and polyamine biosynthesis pathways in Leishmania [43].

In our study, we explored, for the first time, the molecular mechanism of action of *M. nervosa* EO on *Leishamania* sp. Two crucial regulatory pathways were specifically investigated: ergosterol biosynthesis and thiol metabolism pathways. The results revealed that *M. nervosa* EO impacted the leishmanial parasite by modulating multiple cellular processes, including thiol metabolism, which is crucial for maintaining cellular redox balance. 

In fact, to maintain their redox homeostasis, *Leishmania* parasites use the thiol biosynthesis pathway to produce small molecules that neutralize and break down reactive oxygen species (ROS) during their different developmental stages [44]. Interestingly, *M. nervosa* EO showed strong inhibition of the expression levels of all selected genes in this pathway. The alteration initiates at the beginning of the biosynthetic chain, where it inhibits the synthesis of spermidine. This was evident from the under-expression of genes for two catalytic enzymes, namely ornithine decarboxylase (ODC) and spermidine synthetase (SPS). Furthermore, the expression level of the TR gene, which codes for trypanothione reductase, the enzyme responsible for catalyzing the reaction of trypanothione disulfide into trypanothione, was also repressed by the effects of *M. nervosa* EO. In addition, other genes involved in enzyme transcription of trypanothione homeostasis were suppressed. The first enzyme is located in the cytosol and is encoded by the CTP gene, while the second is found in the mitochondria and is encoded by the MTP gene. These two enzymes likely play a crucial role in the scavenging and neutralization of reactive oxygen species (ROS) [45]. These results suggest that the *M. nervosa* EO showed a similar mechanism of action as AmpB in downregulating the thiol metabolism genes of *L. donovani* strains [45]. This provides valuable insights into the potential mechanisms of action responsible for the anti-leishmanial activity of the EO, supporting its potential consideration as an alternative or supplement to conventional antiparasitic agents in combating leishmaniasis. 

In addition, the results of the molecular docking analysis suggest that *t*-cadinol displayed the highest binding energy with the CYP51 target, which is important for the metabolic pathway of ergosterol and crucial in organizing the cytoplasmic membrane of parasites. 

Interestingly, other compounds such as caryophyllene oxide and α-cadinene appear to have the potential to interact with essential amino acid residues in the active site of sterol 14α-demethylase (CYP51) enzyme leading to the effective inhibition of the target enzyme and blocking sterol biosynthesis. Notably, these molecules exhibited low-energy binding, suggesting that these interactions are favorable for the formation of the ligand-receptor complex as reported previously [46]. 

Consequently, the observed effect of *M. nervosa* EO on the parasite cannot be only attributed to its major components. Minor compounds may actively participate in the leishmanicidal potential [6]. The molecular action of *M. nervosa* EO is comparable to that of fluconazole, which primarily targets the CYP51 enzyme (binding energy of −6.9 kcal/mol) by complexation with membrane sterols. Consequently, leakage of the cytoplasmic contents occurred leading to cell lysis and ultimately death of the parasite. 

Regarding α-pinene, the in silico study aligns with previous experimental results, indicating its weak interaction with lanosterol demethylase (binding energy of −5.5 kcal/mol). In fact, a previous study outlined a similar affinity reporting a binding energy of −5.63 kcal/mol [47]. However, it should be noted that the leishmanicidal activity of α-pinene is explained by a different mechanism of action. Specifically, it can modulate the host immune response by inducing an increase in NO levels. Additionally, it exhibits immunomodulatory activity by enhancing phagocytic and lysosomal activity [37]. 

## 4. Materials and Methods

### 4.1. Plant Sampling

*M. nervosa* leaves were collected in Tunisia in April 2019. Authentication of plants was performed by the botanist Pr. Abdelrazzek Smaoui (Biotechnology Center of Borj Cedria) and a voucher specimen was deposited at the CBBC Bioactive Substances Laboratory. 

### 4.2. Essential oils Extraction

The extraction of EO from fresh leaves (150 g) of *M. nervosa* plants was carried out using the Clevenger-type hydrodistillation method for 4 h according to the European Pharmacopoeia technique [48]. The EO was then carefully collected, dried with anhydrous sodium sulfate, and stored at −20 °C in amber glass bottles, until further use. The EO extraction yield was calculated in three independent experiments and expressed as % (*v*/*w*). 

### 4.3. Chemical Analysis of EO Composition

*M. nervosa* EO was analyzed using gas chromatography-mass spectrometry (GC-MS) using an Agilent 7890 GC system (Agilent, Santa Clara, CA, USA), coupled with an Agilent 5975 mass spectroscopy detector (Agilent, Santa Clara, CA, USA) with electron impact ionization (70 eV). The components were separated on an HP-5 MS capillary column (polyethylene glycol: 30 m × 0.25 mm, 0.25 mm film thickness; Agilent Technologies, Hewlett-Packard, Santa Clara, CA, USA). The column temperature was programmed to rise from 40 to 280 °C at a rate of 5 °C/min. The carrier gas was Helium N60 with a flow rate of 1.2 mL/min and a split ratio of 60:1. The scan time and mass range were 1s and 40–300 *m*/*z*, respectively. 

The various constituents were identified by comparison with the software libraries of the Wiley 09 NIST 2011 mass spectral database [36,49].

### 4.4. Antioxidant Activity

The antioxidant activity of *M. nervosa* EO was determined by the DPPH radical scavenging assay and beta-carotene bleaching test, revealing its capacity to inhibit the degradation of beta-carotene and neutralize free radicals. 

#### 4.4.1. DPPH Radical Scavenging Activity

The DPPH radical scavenging activity was evaluated spectrophotometrically according to [50]. In brief, 20 μL of *M. nervosa* EO was mixed vigorously with 980 μL of the DPPH solution (60 × 10^−6^ M) and incubated in darkness for 30 min at room temperature. Then, the absorbance was measured using a UV-Vis spectrophotometer (Synergy, Bioteck, Winooski, VT, USA). The butylated hydroxytoluene (BHT) was used as the reference standard for comparative analysis, and the inhibition free radical DPPH percentage (%) was calculated using the following equation:

Inhibition percentage (%) = [(A_0_ − A_1_)/A_0_] × 100 (1)

A_0_ corresponds to the DPPH solution absorbance without any sample, while A_1_ corresponds to the sample or reference standard absorbance after 30 min of incubation. 

All samples were subjected to triplicate analysis, and the results were expressed in half-maximal inhibitory concentration (IC_50_, μg mL^−1^). Triplicate testing was conducted for all samples. 

#### 4.4.2. Beta-Carotene Bleaching Activity

The β-Carotene bleaching activity of *M. nervosa* EO was assessed as described previously [10,51]. Briefly, 0.5 mg of β-Carotene was dissolved in 1 mL of chloroform. Then, 200 mg of tween 40 and 25 μL of linoleic acid were added to the chloroform solution. The chloroform was removed by vacuum evaporation at 40 °C. Next, 100 mL of hydrogen peroxide was introduced, and the mixture was subjected to vigorous stirring. After preparing the emulsion, 20 μL of *M. nervosa* EO was added to the β-Carotene/linoleic acid mixture in 96-well microtiter plates. Plates were incubated at 50 °C for 120 min, and absorbance was read at t = 0 min and t = 120 min of incubation (automated plate reader ELx 800 Biotek, Biotek, Winooski, VT, USA). 

As a standard reference, the same procedure was carried out using 20 μL of BHT solution (Vigon International, East Stroudsburg, PA, USA) in a solvent. Additionally, a control solution having the same composition, but lacking β-Carotene, was prepared. 

The assessment of antioxidant activity (AA) was performed based on β-Carotene bleaching and the following equation was used:

AA = [(A_t_ − C_t_)/(C_0_ − C_t_)] × 100, (2)

In this equation, A_t_ corresponds to the absorbance value measured for the sample tested after incubation for 120 min, C_t_ represents the absorbance value of the standard reference at the same time point, and C_0_ means the absorbance value of the standard reference measured at the initial time. 

The results are presented in terms of IC_50_ (μg mL^−1^), representing the concentration required to achieve 50% inhibition of β-Carotene bleaching. To ensure accuracy and consistency, all analyses were performed in triplicate. 

### 4.5. Antileishmanial Activity

#### 4.5.1. Parasitic Strains

The in vitroantileishmanial activity of *M. nervosa* EO was assessed against two *leishmania* species *L. major* currently associated with cutaneous leishmaniasis and *L. infantum* linked to visceral leishmaniasis. 

#### 4.5.2. Cultivation of Leishmania Promastigotes

Promastigotes of *L. major* and *L. infantum* were cultured at 27 °C in RPMI medium (Sigma-Aldrich, St. Louis, MI, USA) supplemented with 10% (*v*/*v*) fetal bovine serum (FBS), 100 μg/mL streptomycin and 100 U/mL penicillin. Promastigotes were harvested at the exponential phase of growth and counted in malassez cells. The promastigotes suspension was fixed to a concentration of 2 × 10^5^ parasite/mL [36]. 

#### 4.5.3. In Vitro Antipromastigote Assay

The antiparasitic activity of *M. nervosa* EO against Leishmania promastigotes was performed in 96-well plates. Briefly, serial dilutions of *M. nervosa* EO (concentrations ranging from 7.81 to 500 μg mL^−1^) were added to the parasite suspension at concentrations of 2×10^5^ parasites mL^−1^ in each well. After plate incubation at 27 °C for 72 h, the viability of the parasite was assessed by adding 10 μL of the dye-reduction assay MTT (3-[4, 5-dimethylthiazol-2-yl]-2, 5-diphenyltetrazolium bromide) (SIGMA, Livonia, MI, USA) at a concentration of 10 mg mL^−1^ [52,53]. Finally, 100 μL of DMSO was added to dissolve formazan crystals and the absorbance was determined at 570 nm using a microplate reader (Synergy, Bioteck, Hyderabad, India). Amphotericin B (Sigma-Aldrich, USA) was used as a conventional antileishmanial drug. The IC_50_ (concentration that inhibits 50% of parasite growth) was calculated using Graphpad Prism 5 software. Three replicates were performed for each experiment [36]. 

#### 4.5.4. Antiamastigote Activity

Antiamastigote activity was assessed against macrophages infected with Leishmania promastigotes. Briefly, macrophage cells Raw 264.7 were cultured in RPMI-1640 medium with 10% FBS for 24 h at 37 °C in a humidified 5% CO_2_ incubator atmosphere. Following overnight incubation at 37 °C with 5% CO_2_, the culture medium was removed, and adherent macrophages were infected with stationary-phase promastigotes at a ratio of 10:1 of parasite-macrophage (10 parasites per one macrophage). Then, plates were incubated for 24 h under the same conditions. Non-internalized parasites were discarded by careful PBS washing and infected macrophages were incubated with different concentrations of *M. nervosa* EO (ranging from 0.78 to 200 μg mL^−1^) for an additional 48 h. 

The obtained cultures were fixed with methanol and stained with Giemsa. Examination of stained slides was performed under a light microscope (100×) and the number of intracellular amastigotes was determined in at least 100 macrophages for each sample [54]. Results are expressed as a percentage reduction in infection rate (IR) following treatment with *M. nervosa* EO compared to untreated control as calculated by the following formula:

%IR = 100 − [(infection rate of treated parasites/infection rate of untreated parasites × 100)], (3)

This equation measures the decrease in the rate of infection when treated with *M. nervosa* EO relative to a control group without treatment. 

The calculation of infection rates involved the multiplication of the percentage of infected macrophages by the number of intracellular amastigotes found in each infected macrophage cell and IC_50_ was determined using the GraphPad non-linear regression equation [55]. To determine IC_50_ (half maximal inhibitory concentration), a non-linear regression equation from GraphPad software was utilized [55].

To assess SP (the specificity index), the ratio between IC_50_ values for promastigote and IC_50_ values for amastigote forms was calculated. An SP value between 0.4 and 2, showed that the treatment is effective against both promastigote and amastigote forms. However, for SP values exceeding 2, it suggests that the treatment primarily targets amastigotes rather than promastigotes. Conversely, when SP is below 0.4, it signifies a greater efficacy against promastigotes compared to the amastigote form. These SP values indicate the relative efficacy of the treatment against different stages of the parasite and provide insights into its potential as a therapeutic agent [53]. 

### 4.6. Assessment of Cytotoxicity

The cytotoxicity of *M. nervosa* EO was evaluated on Raw 264.7 murine macrophage cells. Briefly, macrophages were maintained as previously described [53]. Cell viability was determined by microscopic examination and cell counting after staining with 0.1% trypan blue solution. Subsequently, the cell medium was replaced with fresh medium containing various sample concentrations (ranging from 15.125 μg mL^−1^ to 1 mg mL^−1^) and the plates were incubated for an additional 72 h. Viability was assessed using the MTT test and SI (the selectivity index) was calculated as the ratio of IC_50_ macrophage/IC_50_ parasite (53, 36]. The SI on *L. infantum* was determined as the ratio between the LC_50_ values against macrophages and IC_50_ values against promastigote forms. This assessment provided insights into treatment selectivity; with higher SI values indicating a greater preference for inhibiting the parasite without significantly affecting the host macrophage cells. 

### 4.7. Quantitative PCR

The DNA extraction procedure was conducted using the Trizol reagent as outlined in a prior publication [10]. Subsequently, a quantitative PCR (qPCR) assay was conducted with 100 ng of parasite DNA mixed with SYBER Green in 96-well PCR plates as previously described [52]. The specific primers used are: CYP51 (Cytochrome P450 lanosterol 14α-demethylase), ODC (ornithine decarboxylase), SPS (spermidine synthase), TryS (trypanothione synthetase), TR (trypanothione reductase), cytoplasmic tryparedoxin peroxidase (CTP), and mitochondrial tryparedoxin peroxidase (MTP) (Table S1). 

Confirmation of the results involved appropriate melting curve analysis and agarose gel electrophoresis to ensure the accuracy of the PCR products. The results are reported in terms of threshold cycle (Ct) values, automatically computed through the SDS 2.3 sequencing software (Applied Biosystem). Normalization was carried out using an internal control (the 18S rRNA housekeeping gene) [45]. Data analysis followed the 2^−ΔCt^ relative expression method and is presented as the mean ± SD (standard deviation) based on three independent experiments. 

### 4.8. Molecular Docking

#### 4.8.1. Preparation of Target Proteins and Ligands

The crystal structure of the target enzyme sterol 14α-demethylase (CYP51) was prepared from the Protein Data Bank (PDB ID: 3L4D) showing a resolution of 2.75 (https://www.rcsb.org/, accessed on 8 May 2023). To predict the active sites of the target enzyme studied, we used the Computed Atlas for Surface Topography of Proteins (CASTp) tool (http://cast.engr.uic.edu, accessed on 8 May 2023). Additionally, the three-dimensional structures of *M. nervosa* EO compounds α-Pinene and *t*-Cadinol (the ligands), and that of the substrate (fluconazole) were modeled using PubChem Sketcher V2.4 and USCF Chimera 1.17, respectively (Table S2). 

#### 4.8.2. In Silico Study

A molecular docking study was conducted using Vina AutoDock 1.5. 6 tools. For protein preparation, the removal of crystal water molecules and all heteroatoms from the target proteins was achieved through energy minimization. A grid box was established to locate the docking site on the protein target, specifically encompassing the region of interest, notably the active site within the macromolecule (Table S3). Docking was executed to determine a plausible conformation and orientation of the ligand within the binding site with the best conformation chosen based on the lowest binding energy. The resulting docked structures were visualized using PyMOL version 0.99. Additionally, the interaction between the target proteins and ligands was assessed using Biovia Discovery Studio Visualizer v21.1. 0.20298 (Biovia, D. S 2021). This software was available online on 13 December 2022, at https://discover.3ds.com/discovery-studio-visualizer-download. The best pose with the highest score was selected to evaluate the interactions and estimate the binding free energy [46]. 

### 4.9. Statistical Analysis

Analyses were carried out across three independent experiments, with each experiment performed in triplicate. IC_50_ and LC_50_ were calculated using GraphPrism 5 software through logarithmic regression analysis. The mean ± standard deviation (SD) was used to express the values of in vitro antileishmanial activity and in vitro cytotoxicity. Differences were considered significant when *p* < 0.05. 

## 5. Conclusions

In conclusion, *M. nervosa* EO demonstrated notable anti-leishmanial effects, as a potential multitarget agent against Leishmania. It displayed excellent inhibition of gene expression of thiol metabolism pathways and strong potential for molecular interaction with lanosterol demethylase. This enzyme is crucial in the synthesis of membrane ergosterol in parasites. 

The results indicate the potential application of *M. nervosa* EO in the pharmaceutical industry. Further research is needed to examine their efficacy and safety in animal models (in vivo) and to develop combinatorial therapy. Their dual benefits of anti-leishmanial and anti-insecticide activities, as well as their immunomodulatory effects, make them intriguing candidates for further exploration and development as natural remedies for parasitic diseases. 

## Figures and Tables

**Table 1:** GC-MS analysis of *Micromeria nervosa* EO.

N°	Volatils Compounds	Ki*	Ki**	*M. nervosa* (%)
1	α-Pinene	939	1032	26.44 ± 2.55
2	Sabinene	975	1123	0.14 ± 0.07
3	β-pinene	980	1137	1.62 ± 0.53
4	β-myrcene	991	1154	0.29 ± 0.02
5	δ-3-Carene	1011	1159	1.47 ± 0.52
6	*m*-cymene	1027	1178	0.10 ± 0.03
7	*p*-cymene	1027	1180	0.16 ± 0.01
8	*d*-l-limonene	1031	1224	3.25 ± 0.92
9	ɣ-Terpinene	1058	1266	0.10 ± 0.04
10	Oxyde de linalool	1069	1425	0.10 ± 0.02
11	L-Linalool	1098	1553	2.08 ± 0.72
12	α-Campholenal	1123	1592	0.68 ± 0.11
13	*trans*-Pinocarveol	1139	1632	0.73 ± 0.42
14	*cis*-Verbenol	1139	1679	0.51 ± 0.22
15	*p*-Mentha-1,5-dien-8-ol	1172	1738	0.68 ± 0.34
16	4-Terpineol	1178	1740	0.68 ± 0.32
17	L-α-Terpineol	1178	1742	0.41 ± 0.22
18	Carveol	1225	1790	0.21 ± 0.06
19	*trans*-p-Menth-2-ene-1.8 diol	1268	1737	0.88 ± 0.52
20	α-Copaene	1321	1500	1.09 ± 0.13
21	β-Bourbonene	1380	1542	0.15 ± 0.08
22	*trans*-Caryophyllene	1494	1583	1.19 ± 0.19
23	Germacrene D	1510	1732	1.69 ± 0.62
24	α-Cadinene	1524	1752	3.79 ± 0.12
25	δ-Cadinene	1526	1757	1.54 ± 0.14
26	*trans*-Nerolidol	1564	1961	0.97 ± 1.11
27	Caryophyllene oxide	1593	2025	7.73 ± 1.04
28	*t*-Cadinol	1641	2163	26.27 ± 2.82
29	α-Bisabolol	1700	2232	3.77 ± 0.8
	Total			88.92 ± 0.5

Ki* Kovats retention index determined relative to the Retention index of a series of *n*-alkanes (C10–C35) on a HP-5 MS column; Ki** Kovats retention index determined relative to the Retention time of a series of *n*-alkanes (C10–C35) on HP Innowax.

**Table 2:** Antioxidant activity of *M. nervosa* EO.

EO	IC_50_ (μg/mL) ±SD	
	DPPH	β-Carotene
***M. nervosa***	926.33 ± 2.4	489.45 ± 2.7
**BHT**	17.34 ± 0.23	70 ± 5.50

IC_50_: 50% inhibitory concentration. EO: Essential oil. BHT: butylated hydroxytoluene used as a positive control (synthetic antioxidant). The values presented in this table are the mean of three replicates with standard deviation (mean ± SD. *n* =3).

**Table 3:** Antipromastigote activity *M. nervosa* EO.

EO	IC_50_ ± SD (μg/mL)		LC_50_ ± SD (μg/mL)	SI	
	*L. major*	*L. infantum*	Raw 264.7	*L. major*	*L. infantum*
***M. nervosa***	6.79 ± 0.97	5.24 ± 1.64	80.26 ± 3.54	11.82	15.31
**AMB**	0.97 ± 0.08	0.64 ± 0.24	10.62 ± 0.58	10.94	16.59

IC_50_: 50% inhibitory concentration; LC_50_: 50% lethal concentration. The values presented are the mean of three replicates (*n* =3) ± standard deviation (SD). SI: selectivity index calculated as the ratio LC_50_ macrophages/IC_50_ promastigotes. AmpB: amphotericin B.

**Table 4:** Antiamastigote activity of *M. nervosa* EO.

EO	IC_50_ ±SD (μg/mL)		SP	
	*L. major*	*L. infantum*	*L. major*	*L. infantum*
***M. nervosa***	8.04 ± 0.5	7.32 ± 0.87	0.84	0.71
**AmpB**	0.72 ±0.08	0.43 ±0.05	1.34	1.48

IC_50_: 50% inhibitory concentration; LC_50_: 50% lethal concentration. The values presented are the mean of three replicates (*n* =3) ± SD. SP: The specificity index calculated as the ratio of IC_50_ promastigotes/IC_50_ amastigotes. AmpB: amphotericin B.

**Table 5:** Molecular docking data represented in terms of binding energy in Kcal/mole of target enzyme CYP51 from *Leishmania infantum* with fluconazole as substrate and identified ligands, *t*-Cadinol, α-Cadinene, Caryophyllene Oxide, and α-pinene.

Receptors	Ligands	Binding Energy AG(Kcal/Mol)	Active Site Amino Acids	Interaction Type
	***t*-Cadinol**	−7.50	A/ILE. 45, A/PHE. 48, A/MET. 69, A/ILE. 71, A/PRO. 209, A/PHE. 213, A/MET. 357	Alkyl
			A/PHE. 48, A/ILE. 71, A/PHE. 213, A/MET. 357	Pi-Alkyl
			A/ALA. 210	Van der waals
	**α-Cadinene**	−7.30	A/ILE. 45, A/PHE. 48, A/MET. 69, A/ILE. 71, A/ILE. 76, A/PRO. 209, A/PHE. 213	Alkyl
			A/PHE. 48, A/ILE. 71	Pi-Alkyl
			A/PHE. 48	Pi-Segma
			A/GLY. 49	Van der waals
	**Caryophyllene Oxide**	−7.00	A/VAL. 356, A/MET. 357, A/ASN. 455	Conventional Hydrogen Bond
			A/PHE. 48, A/PRO. 52, A/PRO. 209, A/VAL. 356, A/TYR. 456, A/VAL. 461	Alkyl
			A/PRO. 209, A/VAL. 356	Pi-Alkyl
			A/THR. 458, A/MET. 459	Van der waals
	**α-Pinene**	−5.50	A/ILE. 45, A/PHE. 48, A/ILE. 71, A/CYS. 72, A/PHE. 213, A/LEU. 214,	Alkyl
			A/ILE. 45, A/PHE. 48, A/PHE. 213	Pi-Alkyl
			A/GLY. 49, A/ALA. 210	Van der waals
	**Fluconazole**	−6.90	A/ARG. 360, A/VAL. 461	Conventional Hydrogen Bond
			A/MET. 357	Carbon Hydrogen Bond
			A/ILE. 71, A/PRO. 209, A/VAL. 212, A/VAL. 356	Alkyl
			A/MET. 359	Pi-Segma
			A/PHE. 48	Pi-Pi T-shaped
			A/ILE. 45, A/ILE. 76, A/PHE. 213, A/TYR. 456	Van der waals

## Data Availability

Data are contained within the article and Supplementary Materials.

## Supplementary Materials

The following supporting information can be downloaded at: https://www.mdpi.com/article/10.3390/molecules29081876/s1. Figure S1: The active sites of sterol 14-alpha demethylase (CYP51) from *Leishmania*, Table S1: Forward and reverse primers used for qRT-PCR, Table S2: Characteristics and structures of α-Pinene, *t*-Cadinol, Caryophyllene oxide, α-Cadinene and Fluconazol obtained from PubChem and Table S3: Selected Grid parameters for target enzymes.
