# Supplementary material for: Promising Antileishmanial Activity of Micromeria nervosa Essential Oil: In Vitro and In Silico Studies
Source: Molecules. 2024 Apr 19;29(8):1876. doi: 10.3390/molecules29081876 (PMC11055018; doi:10.3390/molecules29081876)
Supplement: Supplementary file 1 [file molecules-29-01876-s001.zip › molecules-2902307-supplementary.pdf]

Supplementary data

Table S1. Forward and reverse primers used for qRT-PCR

Genes	Forward primer	Reverse primer
**CYP51**	GACAACGTCATGGAGGAGATG	CCACCTGGACTGGCTTTAAT
**ODC**	GCACGCGCTTCTCATGAACGTATT	CGAAGAGGATGCAGTTGAAGCTGT
**SPS**	ACTTCTACACGAATGTGCTCCGCA	CATTGCGTACTTGACCGTGGCAAA
**TryS**	TGTCATGAGCGAATGACCAACCGAT	GCTTGCCATTCAACAAACGTCAGGT
**TR**	AATGAGGACGGCTCGAATCACGTT	ATGGCGTAGATGTTGTCCACCGAT
**CTP**	CCAACGGCAGCTTCAAGAAGATCA	TGAAGTCGAGCGGGTAGAAGAAGA
**MTP**	AAGCTAAACACGCAGGTTGTTGCG	TCGATCAGCACACCATAGTCACGA

Table S2. Characteristics and structures of α-Pinene, t-Cadinol, Caryophyllene oxide, α-Cadinene and Fluconazol obtained from Pub Chem

**Sample Name**	**α-Pinene**	**t-Cadinol**	**Caryophyllene oxide**	**α-Cadinene**	**Fluconazole**
**molecular formula**	$C_{10}H_{16}$	$C_{15}H_{26}O$	$C_{15}H_{24}O$	$C_{15}H_{24}$	$C_{13}H_{12}F_2N_6O$
**Canonical SMILES[a]**	CC1=CCC2CC1C2(C)C	CC1=CC2C(CCC(C2CC1)(C)O)C(C)C	CC1(CC2C1CCC3(C(O3)CCC2=C)C)C	CC1=CC2C(CC1)C(=CCC2C(C)C)C	C1=CC(=C(C=C1F)F)C(CN2C=NC=N2)(CN3C=NC=N3)O
**3D Structure[b]**	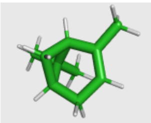	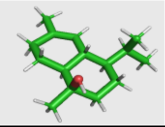	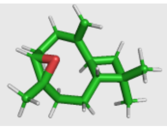	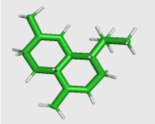	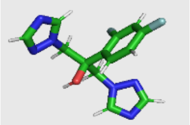

a: PubChem

b :USCF Chimera 1.17.

Table S3. Selected Grid parameters for target enzymes

Protein	Species	PDB ID	Resolution (Å)	Grid Box Center Coordinates			Grid Box Size
				x	y	z	
**Sterol 14-alpha demethylase (CYP51)**	*Leishmania infantum*	3L4D	2.75	32.676	-25.214	-3.329	40 x 40 x 40

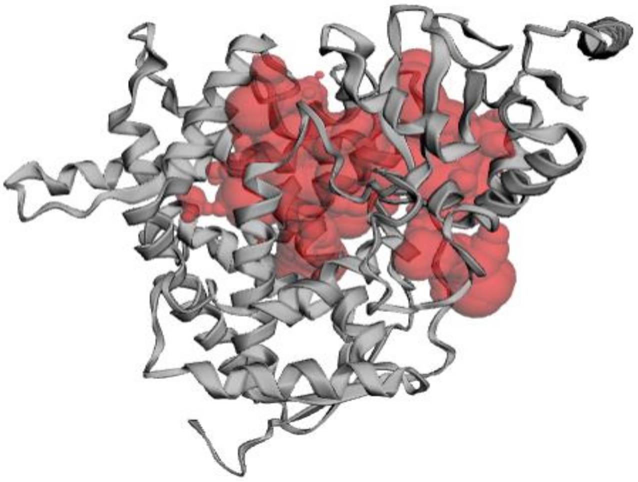

Figure S1. The active sites of sterol 14-alpha demethylase (CYP51) from *Leishmania*

The active amino acid sites of sterol 14-alpha demethylase (CYP51) was predicted using the CASTp server. The CASTp server predicted the binding site amino acids of sterol 14-alpha demethylase (CYP51) from Leishmania. Beside, this enzyme had a pocket ID of an area (SA) Å^{2}of 2295.980 and a volume of (SA) Å^{3}of 1427.802.: ILE.45 , ILE.46 , PHE.48, GLY.49, LYS.50, PRO.52,LEU.53,PHE.55,MET.56, LEU.57, PHE.67,MET.69,ILE.71, CYS.72, ASN.74, ILE.76, VAL.78, PHE.89, ASN.93, SER.97, PRO.98, ARG.99, GLU.100,VAL.101, TYR.102, PHE.104, MET.105,VAL.106, PHE.109,VAL.113, ALA.114, TYR.115, TYR.119, MET.122, ARG.123, GLN.125, LEU.126, ASN.127, LEU.129,GLU.132, LEU.133, LYS.137, PHE.138, ASP.168, SER.171,ALA.172,ILE.174,ILE.175,ALA.178,CYS.181,LEU.182,ALA.197,GLN.198, LEU.200, ALA.201,GLU.204, SER.205, ILE.208,PRO.209,VAL.212,PHE.213, PRO.215, LEU.260,MET.283, ALA.286,ALA.287,MET.288, PHE.289,ALA.290,GLY.291,GLN.292,HIS.293,THR.294,SER.295,THR.296,THR.298, TRP.301, SER.302, HIS.305, GLU.342,ALA.345,ARG.346,GLU.347,SER.348,ILE.349,ARG.350,ARG.351,ASP.352,PRO .353,PRO.354,LEU.355,VAL.356,MET.357,LEU.358,MET.359,ARG.360,LYS.361,ILE.378, ALA.380,PRO.383, LEU.384, LEU.385,HIS.387,GLN.388, PHE.393,PRO.396, ARG.397,GLU.398,ASN.400, MET.405, GLY.414, PHE.415,GLY.416, VAL.419, HIS.420, LYS.421,CYS.422,ILE.423, GLY.424,GLU.425,PHE.427,GLY.428, LEU.429, GLN.431, VAL.432, LYS.433,

LEU.451,GLU.453,PRO.454,TYR.456,HIS.457,THR.458,MET.459,VAL.460, VAL.462 of chain-A.
